# Predicting Axillary Lymph Node Status With a Nomogram Based on Breast Lesion Ultrasound Features: Performance in N1 Breast Cancer Patients

**DOI:** 10.3389/fonc.2020.581321

**Published:** 2020-10-27

**Authors:** Yanwen Luo, Chenyang Zhao, Yuanjing Gao, Mengsu Xiao, Wenbo Li, Jing Zhang, Li Ma, Jing Qin, Yuxin Jiang, Qingli Zhu

**Affiliations:** Department of Ultrasound, Chinese Academy of Medical Sciences and Peking Union Medical College Hospital, Beijing, China

**Keywords:** nomogram, breast cancer, axillary lymph node metastasis, ultrasound, prediction model

## Abstract

**Objective:**

To develop a nomogram for predicting axillary lymph node (ALN) metastases using the breast imaging reporting and data system (BI-RADS) ultrasound lexicon.

**Methods:**

A total of 703 patients from July 2015 to January 2018 were included in this study as a primary cohort for model construction. Moreover, 109 patients including 51 pathologically confirmed N1 patients (TNM staging) and 58 non-metastatic patients were recruited as an external validation cohort from March 2018 to August 2019. Ultrasound images and clinical information of these patients were retrospectively reviewed. The ultrasonic features based on the BI-RADS lexicon were extracted by two radiologists. The features extracted from the primary cohort were used to develop a nomogram using multivariate analysis. Internal and external validations were performed to evaluate the predictive efficacy of the nomogram.

**Results:**

The nomogram was based on two features (size, lesion boundary) and showed an area under the curve of 0.75 (95% confidence interval [CI], 0.70–0.79) in the primary cohort and 0.91 (95% CI, 0.84–0.97) in the external validation cohort; it achieved an 88% sensitivity in N1 patients.

**Conclusion:**

The nomogram based on BI-RADS ultrasonic features can predict breast cancer ALN status with relatively high accuracy. It has potential clinical value in improving the sensitivity and accuracy of the preoperative diagnosis of ALN metastases, especially for N1 patients.

## Introduction

Breast cancer, posing a serious threat to women’s health and social economy, has drawn great attention from researchers for years (1). Axillary lymph node (ALN) status plays an essential role in treatment planning for breast cancer (2), being the most significant prognostic indicator for early stage patients (3). Preoperative staging of ALN status can make a way for optimized clinical decision making. While, currently recognized method for identifying ALN status is sentinel lymph node biopsy (SLNB), which is performed during surgery and requires pathological diagnosis. The SLNB-negative patients would be diagnosed as pN0 in TNM staging (4, 5).

In current clinical practice, axillary ultrasound (US) is commonly recommended for all patients with breast cancer to evaluate ALN status preoperatively (6, 7). However, the SLN cannot be identified by grayscale US, and metastases of isolated tumor cells or micro-metastases are not visible on US. As a consequence, it is difficult for conventional US to achieve high accuracy in identifying axillary nodal metastases. It was reported that US has a sensitivity of 45% to 87% in diagnosing ALN metastases and specificity of 55% to 97% (8). Zhang et al. proved that among N1-3 patients, axillary US had the highest false-negative rate in pathologic N1 patients (9). Hence, it is crucial to improve the preoperative diagnostic accuracy of US in identifying ALN metastases, especially for patients with a minimal number of abnormal nodes.

Previous studies have demonstrated that some ultrasonic features of breast lesions, such as tumor size, margin, and location might be associated with breast cancer nodal metastases and thus can help predict ALN status (10–13). However, in those studies, US findings and tumor clinicopathologic characteristics were simultaneously incorporated to predict ALN metastases (11–13), or a risk model was developed for predicting ALN metastases in a subgroup of patients with invasive ductal carcinoma (10, 11, 13). Considering that the clinicopathologic characteristics, such as histological type, histological grade, and molecular subtype, might directly be related to the probability of ALN metastases, it is necessary to explore the independent contributions of breast lesion US features in determining the likelihood of positive lymph nodes in a preoperative patient population. Therefore, we aimed to construct a predictive model for ALN metastases based on breast lesion US features, to investigate the feasibility of using only US features in identifying nodal metastases preoperatively.

In this study, we summarize the ultrasonic features of the malignant lesions using the breast imaging reporting and data system (BI-RADS) lexicon, the widely accepted standard for defining ultrasonic feature of breast lesions (14). We analyzed the correlations of these ultrasonic features with nodal metastases, developed an ALN metastases predictive model based on these features, and presented it as a nomogram. Such a tool is expected to improve preoperative diagnostic efficacy, especially for N1 patients.

## Materials and Methods

This study is retrospective and was approved by the Institutional Review Board of Perking Union Medical College Hospital.

### Patient Recruitment

A total of 1,024 female patients with breast cancer were enrolled consecutively for model construction and internal validation from July 2015 to January 2018. The clinical data, US images, and pathological results were reviewed. The inclusion and exclusion criteria for establishing the primary and internal validation cohorts were as follows.

Inclusion criteria:

(1) patients pathologically diagnosed as having breast cancer;
(2) ALN status clearly illustrated by pathology after SLNB or ALN dissection (ALND);
(3) breast US scanning performed within one month before surgery;
(4) only a single lesion pathologically identified in each patient, with a diameter less than 5 cm (T1 and T2 stage).

Exclusion criteria:

(1) neoadjuvant chemotherapy or biopsy performed before US scanning;
(2) multiple malignant lesions;
(3) target neoplasms that could not be visualized on US;
(4) incomplete clinical and pathological information.

Finally, a total of 703 consecutive patients were included in this study for model construction and internal validation from July 2015 to January 2018. Then, to validate the efficacy of the prediction model in early breast cancer patients, based on the inclusion and exclusion criteria described above, another 109 patients with pN1/pN0 were recruited at 1:1 ratio as the external validation cohort after primary cohort (From March 2018 to August 2019). Including 51 patients classified as having N1 according to the TNM classification (with one to three metastatic ALN nodes) by postoperative pathology and 58 patients with no ALN metastases (15).

### Clinical and Pathological Information Collection

The clinical and pathological features of the patients, including age, pathological results, and ALN status (LN-positive or LN-negative), were extracted from the medical records.

### Ultrasound Scanning and Imaging Acquisition

All the included patients underwent US scanning before surgery in our Department. Our study did not specify US equipment. The high-quality US images are acquired by four different commercial US devices, which are RS85A (Samsung), IU22 (Philips), Logic 9 (GE) and RS85A (Samsung) with Linear probes (3–12 MHz, centered at 10 MHz). And do not affect the handcrafted extraction of BI-RADS features. The recorded imaging data of the patients were carefully reviewed and selected for further analysis by one experienced radiologist (QZ, 23-year experience in breast US), blinded to the clinical and pathological results. The grayscale and color-Doppler ultrasonic images of both longitudinal section and cross-section were acquired for feature extraction. The largest diameter of each lesion was measured on the grayscale US images.

### BI-RADS-Based US Feature Extraction

Referring to the BI-RADS lexicon and previous researches (16–18), a total of eight ultrasonic features were selected in this study as evaluation indices (**Table 1**). Image reading and feature extraction were conducted by the two radiologists (CZ, 4-year experience in breast US, and YL, 2-year experience in breast US), who were also blinded to the patient**’**s clinical and pathological information. As discrepancies occurred, the agreement would be reached through discussion. Before participating in the study, the two radiologists received systematic training on the BI-RADS lexicon. Inter-observer reliability was assessed by comparing the results of the 2 radiologists in 100 randomly chosen lesions. CZ performed the second feature extraction from 100 randomly selected lesions after 1 week with the same procedure. Then by comparing the results of CZ at two different time points evaluated intra-observer reliability. Finally the inter-observer and intra-observer agreement were measured by kappa statistics.

**Table 1:** Extracted US features.

Feature		Number	Description
Shape	regular	1	A mass that is oval (egg-shaped or elliptical) or round (spherical, ball-shaped).
	irregular	2	Neither oval nor round.
Orientation	horizontal	1	The long axis of the lesion is parallel to the skin line (“wider-than-tall”).
	vertical	2	The anterior-posterior or vertical dimension is greater than the transverse or horizontal dimension (“taller-than-wide”).
Margin	circumscribed	1	The demarcation is well defined and clear, with abrupt transition between the lesion and the surrounding tissue.
	not circumscribed	2	The boundary is poorly defined, and can be characterized as indistinct, angular, microlobulated, or spiculated.
Lesion boundary	abrupt interface	1	The demarcation between the lesion and the surrounding tissue is imperceptible or is a distinct well-defined echogenic rim without any thickness.
	echogenic halo	2	A band bridged by an echogenic transition zone can be perceived.
Echo pattern	hypoechoic	1	The mass has decreased echogenicity compared with fat.
	complex	2	A complex mass containing both anechoic (cystic) and echogenic (solid) components.
Posterior acoustic features	no	1	No shadowing or enhancement is present deep in the mass; the echogenicity of the area immediately behind the mass is not different from that of the adjacent tissue at the same depth.
	enhancement	2	Sound transmission is unimpeded in its passage through the mass. Enhancement appears as a more echogenic (whiter) column deep into the mass. Enhancement is a criterion for cyst diagnosis.
	shadowing	3	Shadowing, i.e., posterior attenuation of acoustic transmission. Sonographically, the area posterior to the mass appears darker.
Calcification	no	1	No calcification.
	macrocalcification	2	Macrocalcifications: coarse calcifications 0.5 mm or greater in size are depicted.
	microcalcification	3	Microcalcifications embedded in the mass are well depicted. The punctate, hyperechoic foci appear conspicuous in a hypoechoic mass.
Vascularity	no	1	Little or No vascularity.
	adjacent	2	present immediately adjacent to lesion
	diffusely increased	3	Diffusely increased vascularity surrounding lesion.

### Model Construction and Validation

The prediction model was built based on multivariate logistic regression analysis. Before construction, multicollinearity analysis was performed by calculating the variance inflation factor (VIF) among the features; a VIF value > 10 was considered to indicate multicollinearity, and the corresponding variables were excluded from the model. All the US features were modeled as categorical data with a dummy variable, adding age as continuous variables, to construct models. In multivariate models, a backward stepwise variable selection procedure was used for model selection based on the Akaike information criterion (AIC). The final model thus built was tested for predictive power using both internal and external validation. Internal validation was performed with the bootstrap resampling method by randomly drawing 500 samples from the primary dataset to avoid overoptimism. The developed model underlying the nomogram was used to predict ALN status of the patients in the external validation cohort. The diagnostic performance of the model in the primary and validation cohorts was evaluated by calculating sensitivity, specificity, positive likelihood ratio, negative likelihood ratio, positive predictive value, and negative predictive value. Receiver operating curves (ROC) and the corresponding area under the curve (AUC) values were used to assess the discriminating ability of the nomogram.

### Statistical Analysis

Statistical analysis was performed using R (http://www.R-project.org) and EmpowerStats software (X&Y Solutions). The variables were compared using Student’s t-test (continuous data) and the Pearson chi-squared test (categorical data). Continuous variables are expressed as the mean ± SD, categorical variables as percentages (%), and p values < 0.05 were considered statistically significant. The degree of intra-observer and inter-observer agreement between the two readers was measured using the κ value, which was interpreted as follows: κ < 0, poor agreement; 0 < κ < 0.20, slight agreement; 0.20 < κ < 0.40, fair agreement; 0.40 < κ < 0.60, moderate agreement; 0.60 < κ < 0.80, substantial agreement; and 0.80 < κ < 1, perfect agreement. The “glm” function was used for the univariate and multivariate logistic regression analyses. The “Hmisc” package was used to plot the nomogram. The “pROC” package was used to plot the ROC curves and measure the AUCs. The “calibration curve” function was used to plot the calibration curves.

## Results

### Clinical Characteristics and Ultrasonic Features of the Primary and External Validation Cohorts

**Table 2** shows the baseline clinical characteristics and ultrasonic features of the 703 patients in the primary cohort and 109 patients in the external validation cohort. A total of 167 (23.9%) patients with ALN metastases were included in the primary cohort and 51 patients (46.8%) with ALN metastases in the external validation cohort.

**Table 2:** Baseline characteristics in the primary and external validation cohorts.

Variable	Primary cohort			External validation cohort		
	Negative for LN metastasis (n = 536)	Positive for LN metastasis(n = 167)	P-value	Negative for LN metastasis (n = 58)	Positive for LN metastasis (n = 51)	P-value
Age	51.3 ± 11.6	50.6 ± 11.4	0.517	51.6 ± 11.5	55.4 ± 11.6	0.088
Size	2.1 ± 0.9	2.7 ± 1.0	<0.001	1.7 ± 0.8	2.5 ± 1.1	<0.001
Shape			<0.001			<0.001
regular	206 (38.4%)	35 (21.0%)		46 (79.3%)	4 (7.8%)	
irregular	330 (61.6%)	132 (79.0%)		12 (20.7%)	47 (92.2%)	
Orientation			0.342			0.002
horizontal	327 (61.0%)	95 (56.9%)		43 (74.1%)	23 (45.1%)	
vertical	209 (39.0%)	72 (43.1%)		15 (25.9%)	28 (54.9%)	
Margin			0.004			<0.001
circumscribed	82 (15.3%)	11 (6.6%)		58 (100.0%)	1 (2.0%)	
not circumscribed	454 (84.7%)	156 (93.4%)		0 (0.0%)	50 (98.0%)	
Lesion boundary			<0.001			<0.001
abrupt interface	327 (61.0%)	40 (24.0%)		55 (94.8%)	12 (23.5%)	
echogenic halo	209 (39.0%)	127 (76.0%)		3 (5.2%)	39 (76.5%)	
Echo pattern			0.333			0.056
hypoechoic	524 (97.8%)	161 (96.4%)		54 (93.1%)	51 (100.0%)	
complex	12 (2.2%)	6 (3.6%)		4 (6.9%)	0 (0.0%)	
Posterior acoustic features			0.054			<0.001
no	396 (73.9%)	109 (65.3%)		39 (67.2%)	24 (47.1%)	
enhance	69 (12.9%)	24 (14.4%)		18 (31.0%)	11 (21.6%)	
decrease	71 (13.2%)	34 (20.4%)		1 (1.7%)	16 (31.4%)	
Calcification			0.531			0.556
no	375 (70.0%)	110 (65.9%)		47 (81.0%)	37 (72.5%)	
macro	6 (1.1%)	3 (1.8%)		2 (3.4%)	2 (3.9%)	
micro	6 (1.1%)	54 (32.3%)		9 (15.5%)	12 (23.5%)	
Vascularity			0.050			0.068
no	210 (39.2%)	48 (28.7%)		21 (36.2%)	13 (25.5%)	
minimal	202 (37.7%)	73 (43.7%)		18 (31.0%)	27 (52.9%)	
abundant	124 (23.1%)	46 (27.5%)		19 (32.8%)	11 (21.6%)	
Histological type			0.163			0.471
invasive ductal carcinoma	396(73.9%)	135(80.8%)		42 (72.4%)	41 (80.4%)	
invasive lobular carcinoma	23(4.3%)	7(4.2%)		9 (15.5%)	5(9.8%)	
ductal carcinoma in situ	101(18.8%)	19(11.4%)		4 (6.9%)	2 (3.9%)	
Others	16(3.0%)	6(3.6%)		3 (5.2%)	3 (5.9%)	
pN status			<0.001			<0.001
pN0	536(100%)	0(0.0%)		58(100%)	(0.0%)	
pN1	0(0.0%)	98(58.7%)		(0.0%)	51(100%)	
pN2	0(0.0%)	29(17.4%)		(0.0%)	(0.0%)	
pN3	0(0.0%)	40(23.9%)		(0.0%)	(0.0%)	

The inter-operator agreements for the ultrasonic features ranged from 0.77 to 0.92 (shape: 0.87; orientation: 0.92; margin: 0.91; lesion boundary: 0.77; echo pattern: 0.92; posterior acoustic features: 0.90; calcification: 0.78; vascularity: 0.81). The intra-operator agreements for the ultrasonic features ranged from 0.79 to 0.96 (shape: 0.91; orientation: 0.94; margin: 0.89; lesion boundary: 0.82; echo pattern: 0.96; posterior acoustic features: 0.91; calcification: 0.85; vascularity: 0.90).

### Diagnostic Performance of the Nomogram

**Figure 1:**
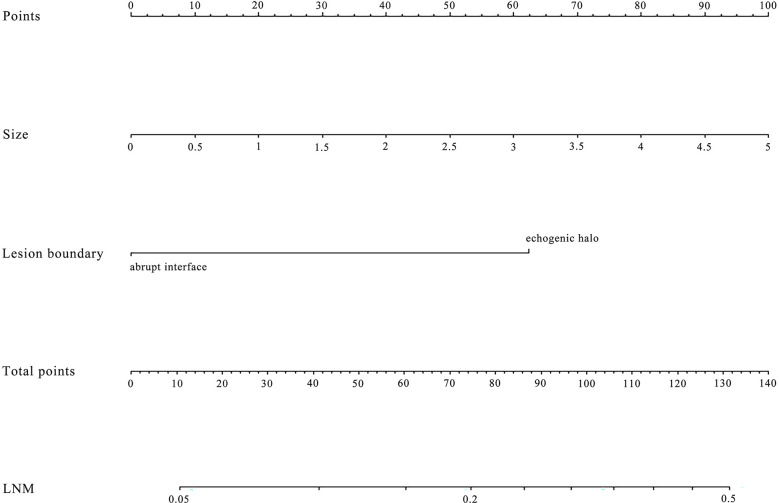
The nomogram was developed in the primary dataset. it included two factors (size, lesion boundary). The nomogram plot provides a visual way to predict the risk of LN metastases for breast cancer patients.

Using multivariate logistic regression analysis, several multivariate models were generated. And after stepwise model selection, two features showed independent correlation with the risk of ALN metastases (**Table 3**) and thus were incorporated into the final nomogram, namely, size and lesion boundary. The nomogram is presented in **Figure 1**.

**Table 3:** Results of Univariate and Multivariate logistic regression analysis in the primary cohort.

Exposure	Univariate analyses	Final multivariate model
Age	1.0 (1.0, 1.0) 0.734	
Size	1.7 (1.4, 2.1) < 0.001	1.7 (1.4, 2.0) < 0.001
Shape		
regular	1.0	
irregular	2.1 (1.3, 3.4) 0.002	
Orientation		
horizontal	1.0	
vertical	1.2 (0.8, 1.7) 0.501	
Margin		
circumscribed	1.0	
not circumscribed	1.6 (0.8, 3.2) 0.169	
Lesion boundary		
abrupt interface	1.0	1.0
echogenic halo	3.7 (2.4, 5.8) < 0.001	4.5 (3.0, 6.7) < 0.001
Echo pattern		
hypoechoic	1.0	
complex	1.5 (0.5, 4.5) 0.434	
Posterior acoustic features		
no	1.0	
enhance	1.1 (0.6, 2.1) 0.651	
decrease	1.4 (0.8, 2.4) 0.249	
Calcification		
no	1.0	
macro	2.2 (0.5, 9.5) 0.285	
micro	1.3 (0.8, 2.0) 0.250	
Vascularity		
no	1.0	
minimal	1.4 (0.9, 2.3) 0.141	
abundant	1.6 (0.9, 2.7) 0.105	

**Figure 2:**
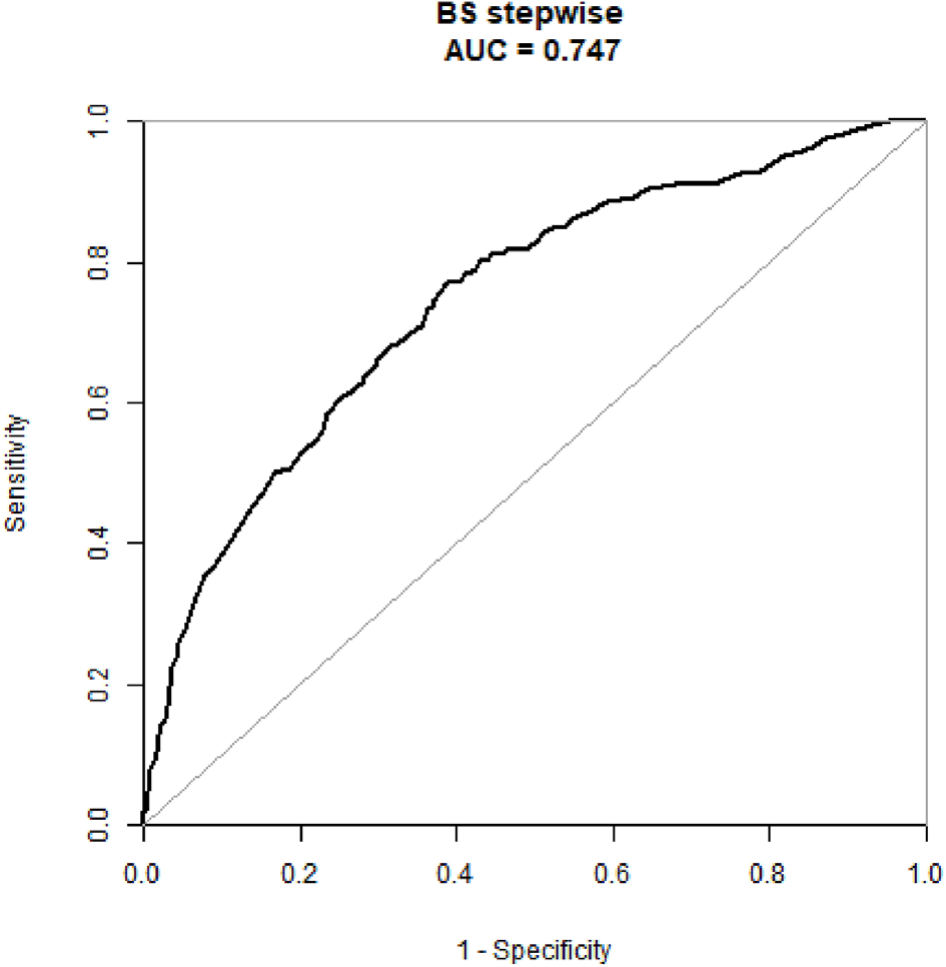
The ROC curves of the prediction model in the primary dataset.

The diagnostic performance of the nomogram in the primary dataset is shown in **Table 4**. The ROC curve of the nomogram showed good predictive power, with an AUC of 0.75 [95% confidence interval (CI), 0.70–0.79] (**Figure 2**).

**Table 4:** Diagnostic performance of the nomogram.

Diagnostic performance	P set	EV Set
AUC	0.7468 (0.7038–0.7898)	0.9065 (0.8424–0.9707)
Specificity	0.6124	0.8966
Sensitivity	0.7711	0.8824
Accuracy	0.6500	0.8899
Positive likelihood ratio	1.9892	8.5294
Negative likelihood ratio	0.3738	0.1312
Positive predictive value	0.3821	0.8824
Negative predictive value	0.8959	0.8966

P, Primary dataset; EV, External validation dataset.

**Figure 3:**
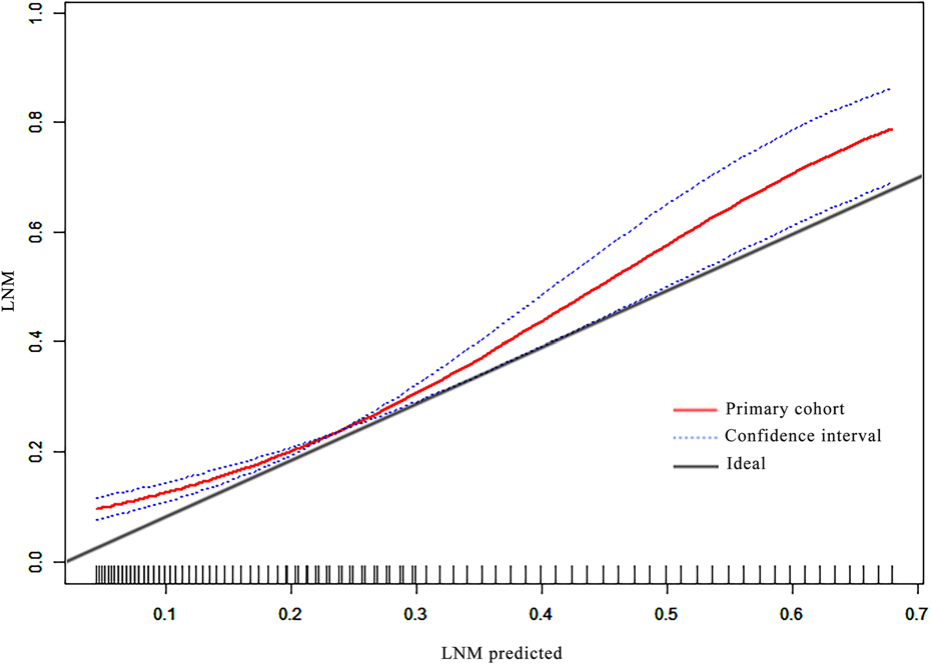
The calibration curves of the nomogram in the primary cohort.

Good calibration was observed for the probability of ALN metastases in the primary cohort (**Figure 3**).

### Nomogram Validation in N1 Patients

**Figure 4:**
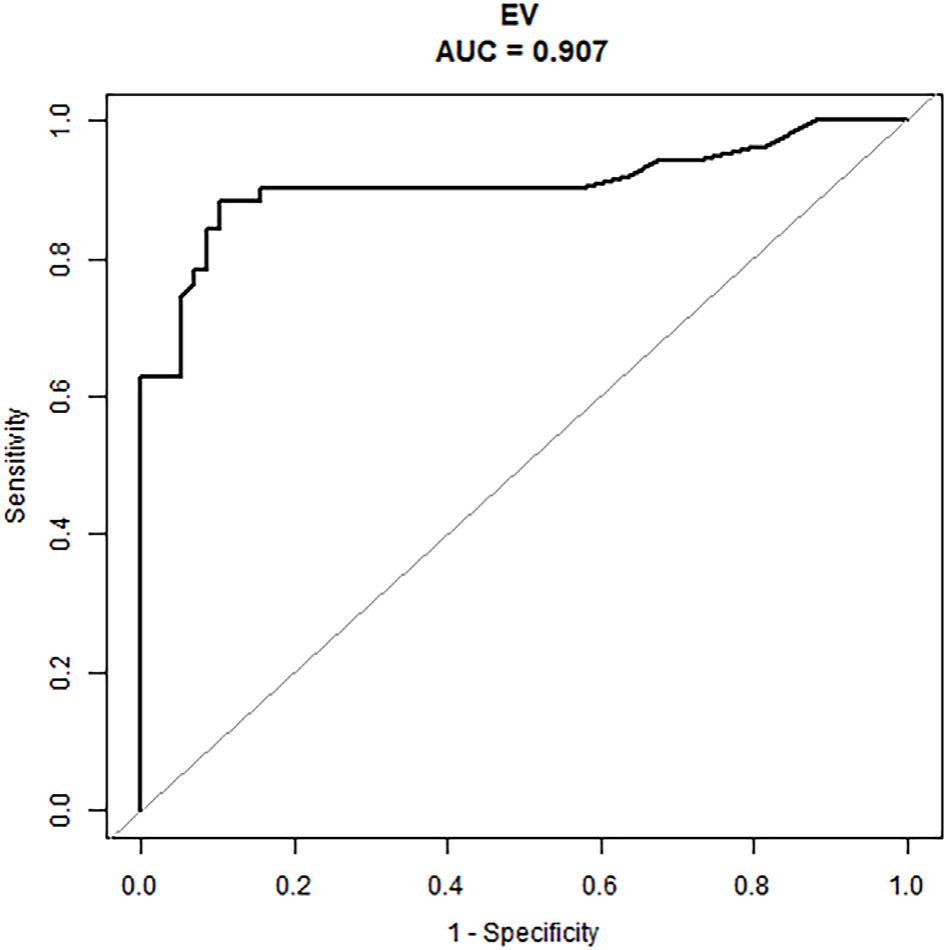
The ROC curve of the prediction model in the N1 patients of the external validation cohort.

An external validation cohort of 109 patients was enrolled using the same criteria used to select the primary cohort and included 51 patients (46.8%) with ALN metastases (the mean number of metastatic ALN nodes was 1.57). The nomogram demonstrated good predictive power (**Table 4**) with an AUC of 0.91 (95% CI: 0.84–0.97) in these N1 patients (**Figure 4**).

## Discussion

Axillary imaging plays an essential role in evaluating ALN status. Axillary US is the primary method for evaluation of axillary nodes, especially in the evaluation of early ALN metastasis. Breast MRI can better demonstrate lymph node metastasis on higher stations (19). However, the use of axillary US in evaluating ALN has been limited by its moderate accuracy and considerable discrepancy among the studies. Some studies have shown that malignant lymph nodes detected by US had a higher node burden than those detected by SLNB, implying a disparity between “ultrasound positive” and “SLNB positive” (20, 21). Moreover, according to previous studies, axillary US tends to perform poorly in identifying metastases in pathologic N1 patients, characterized by one to three abnormal nodes (9). Therefore, to improve the US diagnostic performance for ALN metastases, it is important to improve its accuracy and lower its false-negative rate in N1 patients.

In our study, we developed a prediction model based on BI-RADS ultrasonic features to predict the risk of LN metastases, achieving an accuracy of 65.0% in the primary cohort,and 89.0% in the external validation cohort. A nomogram, incorporating two factors among the lesion US features, showed significant discriminating ability in the primary cohort, and also showed high predictive power in an external validation cohort of early-stage breast cancer patients.

Recent studies have investigated the potential value of ultrasonic images of breast lesions in predicting nodal metastases, with reported AUCs ranging from 0.731 to 0.848 (22–25). Some of these studies showed that US features of breast lesion and axillary lymph nodes are correlated with ALN status (22), and in some studies, high-throughput features of ultrasonic images were proved useful for the prediction of ALN metastases (24, 25). Taken together, these results demonstrate that ultrasonic images of breast lesions can potentially be useful in the preoperative diagnosis of ALN metastases. Considering the nonspecific ultrasonic presentations of metastatic ALNs and the disparity in positive rates between US and SLNB, the images of breast lesions are worth exploring, as they might contain helpful information for the prediction of nodal metastases.

In 2003, a standard protocol for breast US was established in the BI-RADS lexicon and received worldwide recognition (18). The definition and description of the ultrasonic features, the lesion classification, and the reporting system were all clearly defined and illustrated in the lexicon, allowing reliable feature identification. Previous studies have validated clinical-pathological factors and US BI-RADS features of masses could predict breast cancer LN metastasis. Zong et al. (26) suggest that US features of breast mass, like margin, microcalcification, and blood flow signals are significantly correlated with ALN metastasis in early breast cancer. Besides, Guo et al. (12) have proven that irregular shape and high color Doppler flow imaging grades are independent impact factors of ALN metastasis. However, both of them incorporated some clinical-pathological factors simultaneously, like immunohistochemical analysis (ER, PR, Ki-67, and so on) and the histologic grade, which are also highly associated with ALN status. To figure out the independent contributions of breast lesion US features in determining the likelihood of ALN metastasis preoperatively, and to develop a simple and practical nomogram based on US features, we adopted the ultrasonic features defined by the BI-RADS lexicon in 2013 to construct our models (17). A total of eight features were included for modeling, which has been commonly used in differentiating benign and malignant breast lesions. Our results show that some features are also related to ALN status. As shown by the nomogram, tumor size and lesion boundary had more significant impacts on total scores than other features. The prediction model displayed a remarkable ability to predict ALN status, especially in N1 patients, yielding an AUC of 0.901. More importantly, it achieved 88% sensitivity for N1 patients, compared with that in previous studies, which presented false-negative rates as high as 46.2% (9). These results indicate the potential value of our model in increasing sensitivity in the identification of abnormal lymph nodes, as well as in decreasing the rate of preoperatively missed diagnoses, thus bringing benefits to early-stage breast cancer patients.

To note, US readers can predict the probability of ALN metastases associated with the lesion using this nomogram, after routinely extracting the standardized features from the breast lesion ultrasonic images. Apart from its high accuracy, compared with some complex models using additional image processing software, the prediction process used by this model is simple and time-saving. We hope that this model will be widely used in clinical practice as a supplementary to conventional breast US, allowing improved accuracy of preoperative diagnosis of nodal metastases.

Our predictive model has several limitations. First, the sample size of the external cohort was relatively small, and increasing the sample size would be necessary to obtain more convincing results. Moreover, the single-center design of the study might lead to an un recognized bias in patient recruitment, imaging acquisition, and image analysis. Adding data from other medical centers would be helpful in further improving the clinical efficacy of the model.

In this study, a nomogram based on ultrasonic features of breast lesions was developed to predict the risk of ALN metastases in breast cancer patients. The model demonstrated clinical potential in providing a non-invasive, effective, and easy-to-use approach to identify ALN metastases preoperatively, which might aid in clinical decision making.

## Data Availability Statement

The raw data supporting the conclusions of this article will be made available by the authors, without undue reservation.

## Ethics Statement

This retrospective study was approved by the Institutional Review Board of Peking Union Medical College Hospital.

## Author Contributions

YJ and QZ conceived and designed the study. WL, JZ, LM, and MX collected the clinical and image data. YL and JQ performed image pre-processing. CZ and YG analyzed the image data and performed the statistical analysis. YL and CZ wrote the manuscript. All authors contributed to the article and approved the submitted version.

## Funding

This work was supported by CAMS Innovation Fund for Medical Sciences (2017-I2M-1-006).

## Conflict of Interest

The authors declare that the research was conducted in the absence of any commercial or financial relationships that could be construed as a potential conflict of interest.
